# Protective Effect of a Hexapeptide Derived from Rotifer-Specific SCO-Spondin Against Beta-Amyloid Toxicity

**DOI:** 10.3390/ijms26115109

**Published:** 2025-05-26

**Authors:** Zsolt Datki, Rita Sinka, Brian J. Dingmann, Bence Galik, Antal Szabo, Zita Galik-Olah, Gabor K. Toth, Zsolt Bozso

**Affiliations:** 1 Micro-In Vivo Biomolecule Research Laboratory, Competence Centre of the Life Sciences Cluster of the Centre of Excellence for Interdisciplinary Research, Development and Innovation of the University of Szeged, Dugonics ter 13, 6720 Szeged, Hungary; 2 Department of Genetics, Faculty of Science and Informatics, University of Szeged, Közép fasor 52, 6726 Szeged, Hungary; 3 Department of Math Science and Technology, University of Minnesota Crookston, 2900 University Avenue, Crookston, MN 56716, USA; 4 Szentagothai Research Center, Genomic and Bioinformatic Core Facility, 7622 Pecs, Hungary; 5 iBioScience Ltd., 7625 Pécs, Hungary; 6 Department of Medical Chemistry, Albert Szent-Györgyi Medical School, University of Szeged, Semmelweis u. 6, 6725 Szeged, Hungary; toth.gabor@med.u-szeged.hu (G.K.T.); bozso.zsolt@med.u-szeged.hu (Z.B.)

**Keywords:** SCO-spondin, rotifer, biopolymer, beta-amyloid

## Abstract

The Rotimer (rotifer-specific biopolymer) like SCO-spondin (R-SSPO/1), predicted as the main component of this biopolymer, is an adequate base for the design of functional small peptides. This macromolecule is interactive and protective against neurotoxic human-type beta-amyloid 1-42 aggregates (agg-Aβ). The current work presents biological investigations and predictable molecular interaction analysis of DSSNDL and PNCRDGSDE peptides that were synthesized based on the sequences of R-SSPO/1. Viability assays (NADH-dependent cellular reduction capacity, intracellular esterase activity, and motility) were performed on differentiated neuro-type cell cultures (SH-SY5Y and PC12) and on Rotimer-depleted rotifers (*Euchlanis dilatata* and *Lecane bulla*). A control peptide (STTRPTGTT), not found in Rotimer, was also included in the study. All three peptides are present in both rotifer and human proteomes. Among these small molecules, DSSNDL showed a significant protective effect against the toxicity of agg-Aβ both in vitro and in vivo and presumably interacted with its aggregates. The stagogram analysis of amyloid–peptide complexes and the possible bonding competition of these small molecules against aggregation-specific dyes on agg-Aβ surface suggest that DSSNDL affects the properties of these neurotoxic macromolecules. This effective hexapeptide can serve as a promising candidate for further investigations into the inactivation of beta-amyloid toxicity.

## 1. Introduction

In countless cases, natural proteins serve as an initiating point for designing and selecting functional peptide sequences, primarily for industry [1], biomedicine [2,3], or drug development [4].

For neurodegenerative diseases like Alzheimer’s disease (AD), peptides have been designed as active agents derived from natural proteins [5]. Recent advancements in the field of amyloid research have highlighted innovative approaches to counteract amyloid toxicity and aggregation. For instance, the development of peptido-nanocomposites has emerged as a promising strategy in the context of amyloid diseases, offering novel therapeutic avenues by combining the benefits of nanotechnology and peptide design [5]. Additionally, the design of amyloid β-sheet mimics has shown significant potential in antagonizing protein aggregation and reducing amyloid toxicity, providing a molecular framework for the development of effective inhibitors [6]. Furthermore, the application of selected peptidomimetics has demonstrated robust inhibitory effects on amyloid protein aggregation, underscoring their relevance as therapeutic candidates [7]. These approaches collectively contribute to a deeper understanding of amyloid biology and pave the way for the development of targeted interventions against neurodegenerative disorders. Several representative pentapeptides, whose sequences are part of the neurotoxin and aggregation-prone human-type beta-amyloid monomer, are the following: LPFFD [8], LPYFD [9], KLVFF [10], RIIGL [11], GVVIA and RVVIA [12]. Based on their anti-aggregation and beta-sheet breaker effect, these artificial molecules show different levels related to their neuroprotective effect against beta-amyloids. Neurotoxic human-type beta-amyloid 1-42 aggregate (agg-Aβ), a prominent and classical biomarker of Alzheimer’s disease alongside hyperphosphorylated Tau proteins, represents the primary component of senile plaques, signifying localized neuronal death within the brain [13]. Similarly, designs and tests were also based on the amino acid sequences of the SCO-spondin (SSPO) proteins belonging to the thrombospondin family [14]. This conception explains how the neuromodulating peptides WSGWSSCSRSCG and WGPCSVSCG [15] were also created. It is also important to mention that these natural sequences do not always have the desired and assumed biological effect or that not all living models are suitable for their investigation.

It is essential to test the negative effect of agg-Aβ together with the screening of antagonistic molecules, the latter ones that presumably have a protective function in model systems with different profiles. Differentiated forms of various neuron-type monoclonal cell lines, such as SH-SY5Y [16] or PC12 [17], have sufficient neurite arborization to measure the neurotoxic effect of agg-Aβ on them [18]. In this in vitro model, drug candidates with a protective effect against agg-Aβ can be investigated by applying high throughput screening. Deficiencies arising from the limitations of two-dimensional cell culturing (one cell type) can be appropriately compensated with suitable in vivo model systems [19]. Micrometazoans, are adequate animals [20] for cultivating permanently in the laboratory. Rotifers belong to the abovementioned group and are recognized and validated aging [21] and toxicity [22] models. In previous studies, these animals, especially the monogonont (e.g., *Euchlanis dilatata* or *Lecane bulla*) species, proved to be sensitive, fast, simple, and relevant in vivo models [23].

Due to their biological diversity, many protein-like biomolecules of invertebrates and micrometazoans provide an outstanding avenue for drug discovery and development, such as species-specific biopolymers [24]. Due to similar characteristics, rotifers have recently been included in the group of organisms whose biomolecules provide the opportunity to design, synthesize, and conduct exploratory studies of peptide-based active substances [25]. The rotifer-specific biopolymer (Rotimer) [26] plays a central role in the exceptional ability of these animals to catabolize and neutralize human-derived neurotoxic aggregates such as beta-amyloid, alpha-synuclein, and prions [27]. Despite their high sensitivity and indicator properties, rotifers are exceptionally resistant and insensitive to amyloid toxicity [28], but only if they produce a sufficient amount of biopolymer.

The aforementioned aggregated neurotoxin has a lethal effect on Rotimer-depleted animals sensitized by serially induced biopolymer production. The native rotifer biopolymer was protective against agg-Aβ, having anti- and disaggregation properties [29]. The rotifer-specific SCO-spondin (R-SSPO/1) [30], predicted as one of the main components of Rotimer, contains the spondin-type functional domains (e.g., Von Willebrand factor type D domain or low-density lipoprotein receptor class A) that have already been described as specific binding sites for beta-amyloid aggregates in the central nervous system [31].

Spondin-based fibrils (Rotimer or Reissner’s fiber) serve to trap and fix colloidal and micro-structured particles, thus performing a general protective function in rotifers, as well as in vertebrates in their cerebrospinal fluid [32]. Therefore, this protein family is an appropriate choice for designing functional small peptides. It is essential to highlight that the design, selection, and theoretical concept of peptides and their analogs and derivatives require thorough preliminary studies and analysis. The amino acid sequence-based information coded in structure, domains, functions, molecular interactions, isoforms, and genomic backgrounds of the initiating protein should be considered in the design of the sequence of the final peptide molecule [33].

Additionally, there are also physicochemical and biochemical testing methods to detect the interaction of two molecules, such as the stagogram analysis based on molecular crystallization [34] or the competition test based on bonding suppression of special dyes [35]. In this study, we proposed to complement the biological investigations with the aforementioned techniques, which can serve as a starting point and a guide for other researchers in the future. The stagogram analysis is a simple and adequate method for holistic evaluation of the possible interactions between functional peptides and agg-Aβ. The crystallization method, based on optical imaging, has widely been applied primarily in biomarker research [36]. The degree of binding of protein-specific absorbing (e.g., Congo Red and Coomassie Brilliant Blue) [37,38] and fluorescent (Bis-ANS and Thioflavin T) [39,40] dyes to amyloid aggregates, in the presence of a dye-competitor molecule, can illustrate the interacting propensity and possibilities of two relevant molecules.

In the present study, we tested peptides against agg-Aβ-toxicity, derived and synthesized from rotifer-specific biopolymer components, on in vitro and in vivo model systems. Their interaction potentials with this aggregate were also examined.

## 2. Results

### 2.1. The Effect of Short Peptides Derived from Rotifer-Specific Proteins Against agg-Aβ-Toxicity In Vitro and In Vivo

**Figure 1:**
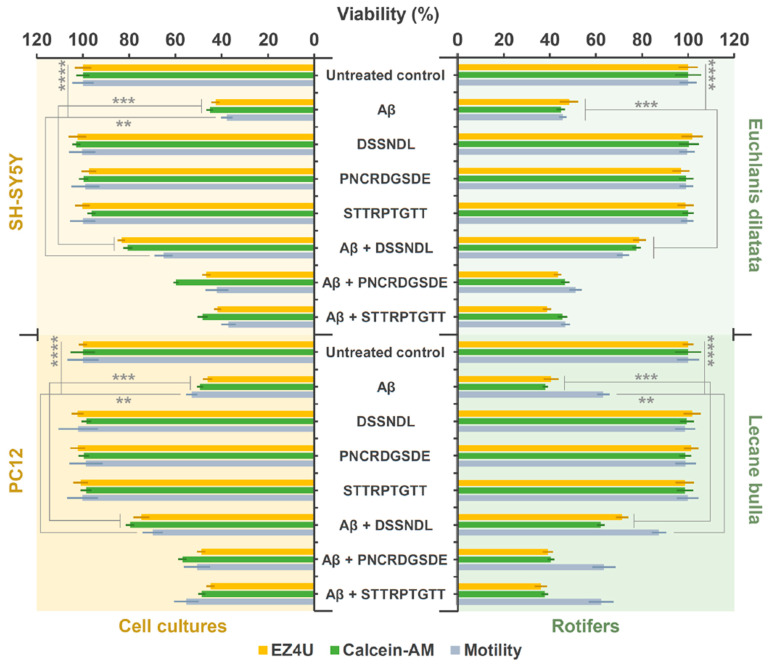
The effect of short peptides derived from rotifer-specific proteins against agg-Aβ-toxicity in differentiated cell cultures (SH-SY5Y and PC12) and biopolymer-depleted rotifers (*E. dilatata* and *L. bulla*). In this study, three different procedures were used to monitor viability. The two specific (NADH-dependent cellular reduction capacity and membrane integrity-dependent intracellular esterase activity) and the holistic (motility detection) profile methods are representative of examining the effect of agg-Aβ (simplified version in the figure: Aβ) and short peptides. The error bars represent SEM. One-way ANOVA with Bonferroni *post hoc* test was used for statistical analysis; the levels of significance are *p* ** ≤ 0.01, *p* *** ≤ 0.001, or *p* **** ≤ 0.0001 (Different number of *, a significant difference from amyloid-treated groups).

In neurodegenerative diseases, especially in AD, one of the main components of the typical amyloid plaques is the neurotoxic biomarker agg-Aβ at different levels [41]. The 42 amino acid version of beta-amyloid [42] can be studied using both in vitro cell cultures and in vivo model organisms (Figure 1). In the present study, differentiated SH-SY5Y and PC12 monoclonal cells were applied, and neurite arborization showed a neuronal character, thus making them suitable for modeling neurodegeneration and cellular toxicity [18]. Rotimer-depleted *E. dilatata* and *L. bulla* monogonont rotifers were used as in vivo models associated with the cell cultures, and they were also used to study agg-Aβ and peptide interactions (DSSNDL, PNCRDGSDE, and STTRPTGTT) designed from rotifer-specific proteins. When designing the peptides, it was important to ensure that the sequences were present in both rotifer and human proteomes, including versions derived from SSPO and non-SSPO sources. Therefore, their selection was based on a comprehensive theoretical analysis. During peptide design, we focused on three key aspects: the presence of functional domains, relevance across multiple species (including rotifers and the human proteome), and hydrophilicity. The peptides DSSNDL, PNCRDGSDE, and STTRPTGTT were synthesized as they fully met these criteria.

The combined results of three viability tests (i.e., NADH-dependent cellular reduction capacity, membrane integrity-dependent intracellular esterase activity, and motility detection) with different profiles reveal the possible antagonistic effects of synthetic small molecules against agg-Aβ. In the case of treatments with active substances, the viability assays showed the same trends for both model systems. Although the measured data and significance levels differ, the effects of agg-Aβ and peptides have the same tendency. The percentage representation of the different methods allows results to be suitably interpreted and compared visually.

The toxic effect of agg-Aβ shows marked significance compared to the untreated control. The examined rotifer-derived peptides were non-toxic or stimulating; however, the DSSNDL hexapeptide significantly reduced the toxic effect of agg-Aβ at all levels. The other two peptides (PNCRDGSDE and STTRPTGTT) were ineffective against the aggregates mentioned. For SH-SY5Y and *E. dilatata*, motility was the most sensitive viability parameter, while for PC12 and *L. bulla*, intracellular processes proved more sensitive.

### 2.2. Suspected Interaction Between agg-Aβ and Short Peptides Derived from Rotifer-Specific Proteins

**Figure 2:**
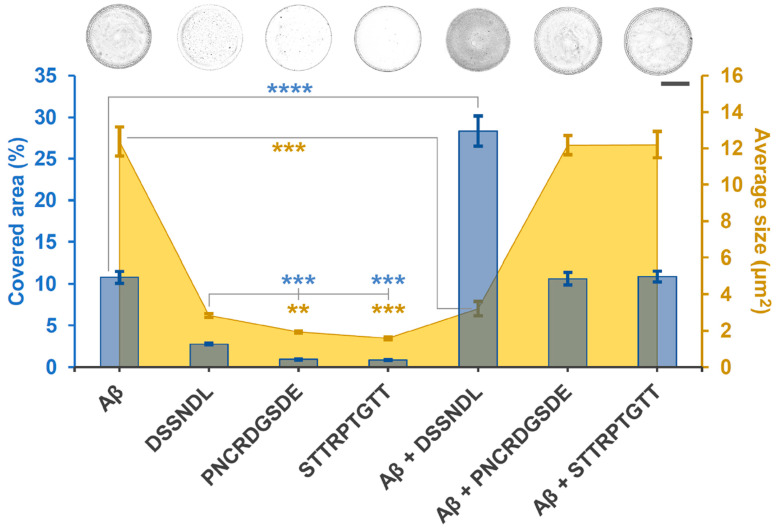
Stagogram-based optical imaging and analysis of the interaction between agg-Aβ and short peptides derived from rotifer-specific proteins. The sizes of the particles of the formed crystals (average size, µM) and the area covered by them (covered area, %) were detected. The simplified version of the agg-Aβ in the figure: Aβ. The error bars represent SEM. One-way ANOVA with the Bonferroni post hoc test was used for statistical analysis; the levels of significance are *p* ** ≤ 0.01, *p* *** ≤ 0.001 or *p* **** ≤ 0.0001 (Different number of *, a significant difference from the group with agg-Aβ alone).

The agg-Aβ effect and peptide counter-effect tested in living systems raised the question of whether there is a supposed interaction between aggregates and investigated small molecules. The first such study to be carried out was a crystallization-based stagogram analysis (Figure 2). This non-biological test made it possible to measure the size of the particles of the droplet pattern obtained by drying the agg-Aβ solution and the area ratio covered by them. It enabled a quantitative correlation and comparison between the manipulated samples; that is, it provided easy-to-interpret parameters for the peptide-aggregate complexes. DSSNDL alone and together with agg-Aβ gave different values from the other samples. The peptide-related area coverage and particle size were significantly higher than those of the other two longer ones, and the peptide-aggregate mixture showed a reverse tendency with agg-Aβ alone. In the drop sample of the agg-Aβ/DSSNDL mix, the area coverage was more significant than in the case of the aggregate alone, and even smaller particles were formed. Overall, we obtained a more homogeneous pattern than the agg-Aβ reference sample. The PNCRDGSDE and STTRPTGTT peptides did not change the pattern of the agg-Aβ stagogram, which is also considered a control macromolecule for them.

### 2.3. Molecular Competition Between Rotifer-Specific Peptides and Amyloid-Specific Dyes

**Figure 3:**
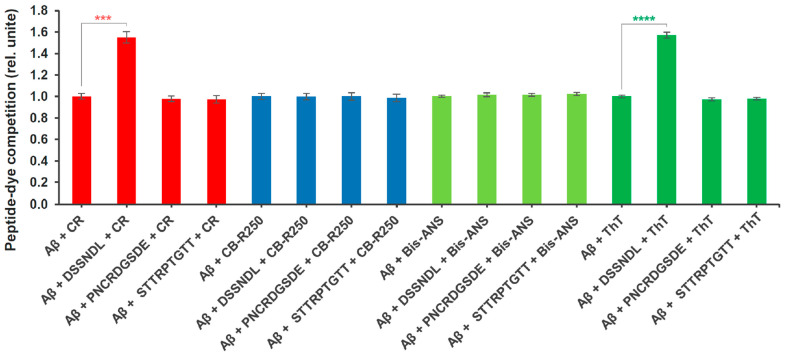
Interacting competition between short peptides derived from rotifer-specific proteins and amyloid-specific dyes. The applied reagents: absorbing dyes were Congo Red (CR) and Coomassie Brilliant Blue R 250 (CB-R250); fluorescents dyes were 4,4′-dianilino-1,1′-binaphthyl-5,5′-disulfonic Acid, dipotassium salt (Bis-ANS), and Thioflavin T (ThT). The degree of competition is the reciprocal (in relative units) of the optical (absorbance and fluorescence) values. The simplified version of the agg-Aβ in the figure: Aβ. The error bars represent SEM. One-way ANOVA with Bonferroni post hoc test was used for statistical analysis; the levels of significance are *p* *** ≤ 0.001 or *p* **** ≤ 0.0001 (Different number of *, a significant difference from groups with agg-Aβ/CR and agg-Aβ/ThT).

The second non-biological assay (Figure 3) is a molecular competition method that shows the binding competition of the tested peptide against a functional dye on the beta-amyloid aggregate. Protein and amyloid-specific dyes (Congo Red, Coomassie Brilliant Blue R 250, Bis-ANS, or Thioflavin T), well accepted in the academic literature, were used to perform this process, which binds to agg-Aβ with high affinity, as well are easily detected. Thioflavin T exhibits the ability to bind to larger oligomers, protofibrils, and fibrils, with a highlighted priority toward larger aggregates [36]. The reciprocal representation of the absorbance and fluorescence values detected at a reduced level shows the relative changes of the peptide-dye competition. These interactions can be evaluated with the initial mixing of agg-Aβ with the relevant short peptides and subsequently testing the binding ability of the dyes. Similarly to the previously described amyloid-dye inhibitory binding results, the DSSNDL peptide showed a difference compared to the reference amyloid-dye control, namely in the case of Congo Red and Thioflavin T. This hexapeptide significantly reduced the binding of both beta-sheet- and fibril-specific dyes to agg-Aβ. The other two peptides did not show any influencing capacity compared to the relevant control samples for any of the dyes. DSSNDL was found to be ineffective as an anti-binding agent against Brilliant Blue R 250 and Bis-ANS. The two absorbent and two fluorescent detection dyes showed no signs of interaction (such as changes in signal intensity or conglomerate formation) with the peptides studied.

The stagogram and the competition measurements provide additional information and provide complementary results to the viability test results as well as a starting point for subsequent investigations.

## 3. Discussion

Investigation of the function, nature, and applicability of biopolymers is a significant field of research in biology nowadays [43]. In recent years, a completely new area within the chemical ecology of rotifers has been established, specifically exploring and studying their external macro-secretory properties related to biopolymer production. In our previous exploratory studies with rotifers, we described a unique phenomenon, namely, that these micrometazoans are able to inactivate and catabolize human neurotoxic aggregates, such as beta-amyloid, alpha-synuclein or prions as a food source [27]. Studying the mechanism of this discovery revealed the ability of rotifers to produce biopolymers, providing them with a protective effect against toxic aggregates [26]. The formation of this biomolecule, namely Rotimer, is a general ability among rotifers. The predicted main components of the Rotimer were rotifer-specific forms belonging to the family of SCO-spondins, especially the R-SSPO/1 spondin protein.

Seeing the anti- and disaggregation effect of the Rotimer as a natural biopolymer [29], a need arose to design peptides with short sequences, the starting point of which was primarily the R-SSPO/1 protein. Our theoretical approach had three main aspects: 1. prioritizing peptide sequences, which contain functional domains that have been described and have validated characteristics; 2. the selected sequence should be relevant in rotifers, in the human proteome, and possibly in other species (Supplementary Table); 3. the peptides prepared should be hydrophilic, i.e., sufficiently water-soluble, to be applicable. The following peptides were designed: DSSNDL, PNCRDGSDE, STTRPTGTT, TEDLENFEYIQSEDFK, CTKTLKMTF, RNIEVNGVE, and IPTDLDYIKV. The first three were synthesized, as they met the conceptual conditions in all respects.

The DSSNDL falls within the Von Willebrand factor type D (VWFD) domain region within R-SSPO/1. This factor-related fiber formation occurs in cultured human-induced pluripotent stem cell-derived cortical neurons with beta-amyloid treatment [44]. In our previous work, we explained the functional similarity of the VWFD-based Reissner’s fibers in vertebrates compared with the external Rotimer fibrils in more detail [30]. What is particularly interesting is that the standard SSPO-based systems exist in species that are evolutionarily distant from each other. In the human proteome, the DSSNDL is found in the immunoglobulin light chain junction region (GenBank: MCH26307.1). A further viewpoint was based on the fact that the epitope region of an anti-SSPO antibody (orb507583) also contains a section (DASNDL) similar to our hexapeptide.

The design of PNCRDGSDE was the most promising as an anti-amyloid molecule. This nonapeptide is located in the low-density lipoprotein (LDL) receptor-like superfamily region of R-SSPO/1. Results described in academic literature reflect that the SSPO-containing LDL region can bind agg-Aβ, whose accumulation in the central nervous system contributes to the formation of senile plaques, a morphological hallmark of AD [45,46,47]. In addition, our peptide contains the DGSDE segment, which has already been studied in itself [14] or as an active agent [15], starting from human SSPO. The length of the PNCRDGSDE peptide segment was determined based on its occurrence in rotifer species and human proteins, which is a common denominator.

The STTRPTGTT is also a nonapeptide but not an SSPO sequence. It occurs several times [30] in previously published proteomic studies (rf 1 ctg.003923F.g152291.t1; rf 1 ctg.000390F.g73244.t2; rf 1 ctg.000390F.g73244.t1; rf 1 ctg.003923F.g136261.t1), in multiple copies in each of them. These uncharacterized proteins could only be identified in the case of *E. dilatata*; therefore, they cannot form the main component of Rotimer, which is universally found among rotifers. Since these disordered molecules were detected in all samples of the biopolymer conglomerates, STTRPTGTT may have a specific effect on these small animals. This sequence is also characteristic of the Sodefrin precursor repeats C in rotifers [48] and is also a peptide pheromone in invertebrates [49,50].

The peptides discussed above were designed to prevent the toxic effects of agg-Aβ on cell cultures and sensitized Rotimer-depleted rotifers (*E. dilatata* and *L. bulla*). These two differentiated cell types (SH-SY5Y and PC12) are widely accepted as in vitro neuronal models since they are monocultures capable of neurite arborization, showing sufficiently high sensitivity, e.g., for treatments with agg-Aβ. Relatively high doses of agg-Aβ were used in the viability tests to achieve sufficient toxicity. Oligomers are known to be more toxic than protofibrils or fibrils [51,52]. However, it was also shown [53,54] that the aqueous solution of agg-Aβ contains low and high-organized aggregates in the micromolar range; the entire spectrum is present in a suitable equilibrium state. All aggregation levels can be found in the sample created during a 3-day aggregation process [55,56,57,58]. Our research group and collaborators have extensive experience in synthesizing, characterizing, and preparing agg-Aβ or related derivatives [59,60,61]. The dose of the agg-Aβ sample needs to be appropriate for biological and non-biological measurements due to the joint interpretability of the results. In the sedimentation-based tests (interaction and competition), it was essential to have colloidal-sized aggregates in the samples and to achieve sufficient biological toxicity in parallel with this criterion. During this dose-based synchronization, the effect of the investigated relevant peptide against agg-Aβ became measurable and comparable.

After designing peptides based on rotifer-related proteins and optimizing agg-Aβ preparation, we conducted both biological and non-biological tests. The exceptional catabolic ability of rotifers in the field of neurotoxic aggregates, as well as the protective effect of their biopolymer against them, made a conceptual starting point to investigate the possible protective effect of derived peptides against agg-Aβ. In the present work, two biological models at different levels (in vitro and in vivo) were used, with a total of two differentiated cell types (SH-SY5Y and PC12) and two microscopic animal species (*E. dilatata* and *L. bulla*). Three different physiological parameters (i.e., NADH-dependent cellular reduction capacity, membrane integrity-dependent intracellular esterase activity, and motility detection) were detected in these living systems. The models, the measurements performed on them, and their theoretical cross-sections sufficiently cover the first-level exploratory studies necessary for the primary determination of the investigated peptides concerning agg-Aβ interactions. The toxic effect of agg-Aβ on intracellular functioning suggests that it can damage cultures as well as live animals (with reduced Rotimer content) on the cell level. The decrease in cell movement of in vitro cultures and the swimming speed of rotifers can probably be explained by the negative modulating effect of this well-known neurotoxin. The universal protective effect of the DSSNDL peptide prevents the development of these drastic processes, probably through its molecular interactions in the relevant samples. This peptide was able to significantly inactivate the effect of agg-Aβ to a certain extent, both in vitro and in vivo, which may indicate that it does not primarily exert its protective influence on the model system but interacts with the applied aggregated macromolecules.

The viability results were supplemented with molecular interaction studies. For one of the non-biological tests, a stagogram analysis was applied (Figure 2), a widely accepted indicative method for displaying different sample properties and interaction ability based on their optical diversity [36,62]. Despite its simplicity, this technique is a sensitive crystallization procedure, where the size of the formed particles and their areal coverage are detected and the formed droplets analyzed. Furthermore, even the dried samples can be qualitatively distinguished. The limited but precisely defined chemical environment with few components is easily evaluated and, thus, an excellent in vitro system. The essence of this drying process is the presence of influencing molecules and ions, their absence, or differences in their amounts. The groups with different compositions were examined and compared with the corresponding controls. In this case, the reference components were the agg-Aβ alone and peptides (DSSNDL, PNCRDGSDE, and STTRPTGTT) in contrast to amyloid–peptide complexes. The groups of the crystallized droplets differed in density and structural characteristics. The profile of the analyzed samples refers to the interactions between the components in them during the drying process. Based on the optical markers of the stagogram, it can be established that the phenotypes of agg-Aβ, peptides, and their complexes significantly differed from each other. The fact that DSSNDL itself was distinct from the other two peptides suggests that it has different chemical properties. Together, agg-Aβ/DSSNDL showed a denser pattern compared to the other mixed variants. Based on the results, it can be stated that there is a supposed molecular interaction between agg-Aβ and the DSSNDL hexapeptide. The other two investigated peptides are unlikely to bind to agg-Aβ, as the pattern of their sedimented sample by centrifugation did not differ from the reference control.

Another version of chemical-type non-biological measurements is based on binding site-related competition of the investigated molecules. The mechanism of colored (Congo Red and Coomassie Brilliant Blue R 250) or fluorescent (Bis-ANS and Thioflavin T) dyes that bind to beta-amyloid protofibrils and fibrils with high affinity is known [63]; thus, changes in the binding characteristics within a given system can be informative regarding the properties of other agents through these interactions. The agg-Aβ separated by centrifugation-based sedimentation, can carry small peptides that are stably bound to it, and the molecular coating formed by these interactions can prevent the dyes above from fitting into the actual aggregates. During the very short (a few minutes) incubation time of the molecules, it is unlikely that either the peptides or the dyes could trigger disaggregation in the case of agg-Aβ. Based on our knowledge, it can be assumed that most of the small binding molecules find suitable binding/interaction points on the surface of agg-Aβ. Of the three investigated peptides, the DSSNDL was also the one in this case, which resulted in a significant signal reduction when measuring the dyes; that is, it prevented their binding to agg-Aβ with increasing competitive interactions. The fact that this hexapeptide only competed with dyes that prefer beta-sheet regions (Congo Red and Thioflavin T) is worth highlighting, and the assumption is that it prefers mutual conformational surfaces.

The measurements and results obtained in the present work are sufficient to conclude that the R-SSPO/1-derived DSSNDL peptide has a protective effect against agg-Aβ toxicity and that it likely exerts this through binding to aggregates. The current data can serve as a basis for investigating further mechanisms of action and for the possible application of DSSNDL in translational biomedicine or drug development against neurodegenerative diseases. Beyond that, this outstanding hexapeptide can equal the protective potential of peptides (LPFFD [5], KLVFF [7], and RIIGL [8]) previously designed as protective agents against beta-amyloids, yet in this case, it conceptually differs from them. The novelty of DSSNDL lies in the fact that this molecule was created as a translation of an evolutionary relationship and was not derived from the beta-amyloid peptide sequence.

## 4. Materials and Methods

### 4.1. Materials

Materials applied in this work were the following: algae (*Chlorella vulgaris*; BioMenu, Caleido IT-Outsource Kft.; cat. no.:18255); yeast (*Saccharomyces cerevisiae*; EU-standard instant granulated form, cat. no.: 2-01-420674/001-Z12180/HU); dyes were obtained from Sigma-Aldrich: Congo Red (cat. no.: 75768), calcein-AM (cat. no.: 17783), thioflavin T (ThT; cat. no.: T3516) and 4,4′-dianilino-1,1′-binaphthyl-5,5′-disulfonic acid dipotassium salt (Bis-ANS, cat. no.: D4162); from Reanal: Coomassie Brilliant Blue R 250 (cat. no.: 03144, Reanal, Budapest, Hungary); from Corning: surface-treated 96-well plate (cat.no.: 3695, Costar, Corning Inc., Union City, CA, USA), non-treated (cat. no.: 430591) Petri dishes and surface-treated culture flasks (cat. no.: 430168); from Greiner: 96-well microplates with half-area (cat. no.: 675101) and 384-well microplates (cat. no.: 781061, Greiner Bio-One International, Kremsmünster, Austria); from Biomedica EZ4U (cat. no.: BI-5000, Medizinprodukte, Wien, Austria); from Merck: distilled water (DW; Millipore Ultrapure); universal plastic (nylon) web for food preparation and rotifer isolation (pore diameters: 10 and 50 µm); standard culture medium (mg/L): Ca^2+^ 37; Mg^2+^ 17; Na^+^ 2; K^+^ 1; HCO_3_^−^ 180; SO_4_^−^ 4; Cl^−^ 1; NO_3_^−^ 2; SiO_2_ 6, with a total content of 250 mg/L, pH = 7, and conductivity (23 °C) between 390–398 µS/cm. Human-type beta-amyloid 1-42 (cat. no.: A14075, human Amyloid b-Peptide 1-42; cat. no.: 107761-42-2) was purchased from AdooQ Bioscience LLC., Irvine, CA, USA. Tentagel S RAM resin was obtained from Rapp Polymera GmbH, Tübingen, Germany. Diisopropyl carbodiimide and Ethyl-2-cyano-2-(hydroxyimino)acetate were from Fluorochem, Hadfield, UK. *N*^α^-Fmoc-protected amino acids and *N*-methyl-2-pyrrolidone were purchased from Iris Biotech GmbH, Marktredwitz, Germany. Piperazine was from Alfa Aesar GMbH & Co KG, Karlsruhe, Germany, and ethanol was from Reanal Laboratory Chemicals, Budapest, Hungary. Dithiothreitol and triisopropylsilane were obtained from abcr GmbH & Co KG, Karlsruhe, Germany. *N*,*N*-dimethylformamide was from Merck KGaA, Darmstadt, Germany, and trifluoroacetic acid was from VWR International, Debrecen, Hungary.

### 4.2. Rotifer-Specific Peptide Synthesis

The peptides based on R-SSPO/1 (rf 1 ctg.000390F.g65781.t1, MW: 321850): DSSNDL (position: 1119–1124) and PNCRDGSDE (position: 1414–1422), and the uncharacterized rotifer-specific protein-based (rf 1 ctg.003923F.g152291.t1, MW: 284572) peptide STTRPTGTT (with 18 occurrence points in the protein) were synthesized (Supplementary Figure S1). These C-terminal amidated short peptides were synthesized on a TentaGel S RAM resin using *N*^α^-Fmoc-protected amino acids with a CEM Liberty Blue microwave peptide synthesizer (Matthews, NC, USA). Activations were carried out with diisopropyl carbodiimide and Ethyl-2-cyano-2-(hydroxyimino)acetate in *N*,*N*-dimethylformamide. Removal of Fmoc groups was undertaken with a 10% piperazine/10% ethanol in *N*-methyl-2-pyrrolidone mixture. Peptides were cleaved from the resin using a 90% trifluoroacetic acid, 4% water, 3% triisopropylsilane, 3% dithiothreitol cocktail and purified on a RP-HPLC system [64]. The purity of the peptides was analyzed by HPLC and was at least 99%. Molecular weight of the peptides was determined by ESI-MS (STTRPTGTT, calculated: 919.97, measured: 919.9; PNCRDGSDE, calculated: 990.98, measured: 991.0; DSSNDL, calculated: 648.6, measured:648.8). The HPLC chromatogram and ESI-MS spectrum of these purified peptides derived from rotifer-specific proteins are shown in Supplementary Figure S2.

### 4.3. Preparation of the Amyloid Aggregates

The preparation of amyloid peptides, which are highly prone to aggregation and thus assemble into macromolecules, is usually a complex procedure; however, in the present case, already validated and standardized steps have been applied [55,65,66]. The dose of the aggregated human-type beta-amyloid 1-42 stock solutions was 1 mg/mL in DW. The aggregation time was 72 h at 23 °C (pH 3.5). The neutralization (to pH 7) was performed with NaOH (1 M) [24]. At the end of the aggregation process, the samples were vortexed (3 min; 550 rpm), and before using them, they were ultrasonicated (Emmi-40 HC, EMAG AG, Herford, Germany) for 15 min at 45 kHz to achieve homogenization and semi-sterilization. This sample form aligns with that applied in our previous works and contains both smaller and larger structural forms of the peptide aggregates. The final concentration of agg-Aβ (diluted with standard media) was 20 µM in viability-related experiments and 200 µM (diluted with DW) in non-biological assays. A larger beta-amyloid dose is necessary for molecular interaction and competition studies due to the amount to be centrifuged.

### 4.4. In Vitro and In Vivo Cultures

Both in vitro and in vivo models provide valuable platforms to investigate agg-Aβ, its aggregation process, and the potential of antagonistic molecules, such as short peptides, which attenuate cell-toxic processes. In vitro models, utilizing validated and standardized protocols, included the differentiated SH-SY5Y human neuroblastoma and the equally differentiated PC12 adrenal pheochromocytoma cell types, which originate from cultures maintained at our institute.

The in vivo experiments were performed on *E. dilatata* and *L. bulla* monogonont rotifer species; therefore, no specific ethical permission was needed according to the relevant international regulations. The species have been maintained and cultured at standard laboratory conditions for several years. The culturing details and the history of the abovementioned rotifers were precisely described previously by Datki et al. [26]. For standard food cultures, a mixture (0.6 mg/mL) of homogenized baker’s yeast and alga (1:1 ratio) was used after heat-inactivation and filtration (diameter of particles ranged 8–10 µm).

### 4.5. Viability Assays

The toxicity of agg-Aβ and the effect of potential antagonistic peptides were investigated (Figure 1) using three procedures with entirely different profiles (two specific and one holistic). The agg-Aβ (200 µM) and newly synthesized peptides (100 µM) were mixed for 30 min by vortexing every 5 min. The final treatment concentration on cell cultures or rotifers was 10× dilution of the amyloid–peptide mixture (20:10 µM). The treatment time was 48 h on cells and 24 h on rotifers in all viability experiments. First, the NADH-dependent cellular reduction capacity was examined, where the cells (SH-SY5Y and PC12) were cultured on a surface-treated 96-well plate (n = 12 well in each group) in a confluent monolayer pattern (cell number was 205 ± 14 in one well). The monogonont rotifers (n = 12 wells in each group; *E. dilatata*: 80 ± 9 and *L. bulla*: 110 ± 15 entities/well) were treated in 96-well plates (with half area), having a nonpolar surface. The mitochondrial and cytoplasmic reduction capacity was detected with the special EZ4U kit, where the soluble formazan, produced after 4 h of incubation (absorbance: 490/630 nm), is proportional to the viability of the cells in the animals.

The cellular membrane integrity of the relevant biological model was detected under conditions consistent with the above measurement, where the intracellular esterase activity was detected using calcein-AM fluorophore. The fluorescence intensity (ex/em: 490/520 nm) is also directly proportional to the viability of the cells and rotifers.

In the motility-based comprehensive viability test, cell movement (SH-SY5Y: 66 ± 10 and PC12: 52 ± 12 μm/h) and rotifer swimming speed (*E. dilatata*: 470 ± 60 and *L. bulla*: 264 ± 44 μm/s) were investigated. After the targeted treatment, the cells were monitored for 6 h in a surface-treated 96-well plate (n = 12 well in each group; 4–5 cells/well; with time-lapse recordings every 5 min), while the rotifers were monitored in a non-treated 96-well plate with half area (n = 12 one-housed entities, one rotifer/well; 3-min continuous video recording at the end of the treatment period).

### 4.6. Derived Short Peptide-Amyloid Interaction Assay Based on Stagogram Optical Analysis

One of the easiest methods of examining the presence or absence of interactions between two molecules is the optical analysis of stagograms, based on the drying and crystallization of sample mixtures (Figure 2). After 30 min of incubation (applying vortex in 5-min intervals), the interaction between synthesized peptides (200 µM) and beta-amyloid aggregates (100 µM) was investigated. This mixture has a high material content with a colloidal size range. After centrifuging (38,000 g/30 min) and discarding the supernatant, the pellet was resuspended in 20 µL DW.

Drops (n = 10) with 1.5 µL volume were put onto the non-treated hydrophobic plastic surface of a Petri dish; then, they were dried for 2 h at 45–50% humidity. The formed stagograms of the drops were detected by light microscopy (Leitz, Labovert, Germany) and were photographed (Nikon D5600, Japan). The Image-J evaluation process used here is as previously described [29], in which relevant data of the conglomerate-covered area (%) and their particles’ average size (µm^2^) were analyzed.

### 4.7. Binding Competition of Derived Short Peptides with Amyloid-Specific Dyes

One of the basic testing methods of competition between molecules is measuring the marking capacity of target molecule-specific profile dyes (Figure 3). The creation of the pellet of the peptide–amyloid mix is entirely consistent with the steps of the stagogram methodology. The pellet, resuspended with DW, was mixed with the given dye (Congo Red, Coomassie Brilliant blue R 250, Bis-ANS, or Thioflavin T) for 5 min. These samples were centrifuged again (38,000 g for 30 min), and then the pellet was resuspended in DW (400 µL in each dye-related sample). The detection was carried out in a 384-well plate (n = 12 wells/sample type; 30 µL/well) and was recorded with a FLUOstar Optima microplate reader (BMG Labtech, Ortenberg, Germany). The detection parameters of the dyes were as follows (nm): 540 abs of Congo Red (10 µM), 590 abs of Coomassie Brilliant Blue R 250 (10 µM), 405/520 ex/em of Bis-ANS (5 µM) and 450/480 ex/em of Thioflavin T (5 µM). The number of laser flashes per well was 25. Calibration/gain adjustment was 1% of the maximal relative intensity of fluorescence.

For synchronization of different fluorophore data, the percentage value of absorbance and fluorescence is represented in reciprocal form. The peptides reduce the binding of the dyes, thereby increasing the measured optical values as well; however, the degree of competition increases. It was not the measured value but the extent of the competition effect that was analyzed.

### 4.8. Statistics

Statistical analysis was performed with SPSS 23.0 (SPSS Inc., Chicago, IL, USA) and GraphPad Prism 7.0b software (GraphPad Software Inc., La Jolla, CA, USA) using one-way ANOVA with Bonferroni post hoc test. A 99% confidence level (1-alpha = 0.01) was used in parallel with the applied statistics, and a 1% error was handled. The homogeneity of variances was controlled; however, the empirical data were parametric and found optimal for the Bonferroni post hoc test. The significance variable levels are *p* ** ≤ 0.01; *p* *** ≤ 0.001 and *p* **** ≤ 0.0001. The error bars show the standard error of the mean (SEM). All relevant and significant relations and comparisons between the affected groups are defined in the given figure legend.

## 5. Conclusions

Various interactions between neurodegeneration-specific amyloid and other small molecules of biological and synthetic origin have been investigated in the literature. The present work aims to investigate the inactivation and molecular interactions of agg-Aβ with different synthesized peptides. Peptides DSSNDL and PNCRDGSDE, derived from the sequence of the R-SSPO/1 protein, were predicted as components of the rotifer-specific biopolymer. A segment of the rotifer-specific protein, associated with Rotimer, was also used as a control peptide (STTRPTGTT). The exploration into the exceptional ability of rotifers to catabolize neurotoxic beta-amyloid, alpha-synuclein, and prions, and all these aggregates with the help of Rotimer, led to the design of the above peptides.

Further theoretical selection and planning of the peptide sequences were made with detailed consideration of genomic and proteomic knowledge, such as transcriptome occurrences and identities in different species or beta-amyloid binding sites on functional domain regions characteristic of SCO-spondins. The agg-Aβ/peptide interactions were tested on biological (cell cultures and micrometazoans) and biochemical (crystallization stagogram pattern and dye suppression competition) model systems, investigating toxicity and protective effects as well as aggregate and small molecule interactions. The results demonstrate that DSSNDL hexapeptide can significantly reduce the toxic effect of agg-Aβ on cells and organisms and modulate its physicochemical characteristics and dye binding capacity. It can be assumed that this small peptide exerts its effect primarily by interacting with agg-Aβ, probably with a preference for beta-sheet structural domains.

The novelty of the work is the derivation of amino acid sequences encoded by rotifer-specific biopolymer proteins into peptides and their translational application for the inactivation of neurodegenerative-type beta-amyloid aggregates. The relatively hydrophilic nature of DSSNDL, STTRPTGTT, and PNCRDGSDE, in contrast to known beta-sheet breakers or Aβ-binding agents, such as LPFFD, KLVFF, and RIIGL, may suggest an alternative mechanism of action for DSSNDL. To further elucidate this mechanism, future studies (e.g., FTIR or circular dichroism) should investigate the conformational properties of these peptides. This will provide deeper insights into their interactions with beta-amyloid and their potential as therapeutic agents.

## Data Availability

The raw data supporting the conclusions of this article will be made available by the authors on request.

## Supplementary Materials

The following supporting information can be downloaded at: https://www.mdpi.com/article/10.3390/ijms26115109/s1.
