# Supplementary material for: Protective Effect of a Hexapeptide Derived from Rotifer-Specific SCO-Spondin Against Beta-Amyloid Toxicity
Source: Int J Mol Sci. 2025 May 26;26(11):5109. doi: 10.3390/ijms26115109 (PMC12154537; doi:10.3390/ijms26115109)
Supplement: Supplementary file 1 [file ijms-26-05109-s001.zip › Suppl Figure S1.pdf]

R-SSPO/1

rf 1 ctg.000390F.g65781.t1

MW: 321850

(Datki et al., 2023)

MGSIPNLFRFILTVIIVISSNQALLRADEVEHSEEHFCDKVEKNNDVCPTPSKFDILSEIDKEDKEWKIDFEQFKKEKITS
SSELKNEISSRFNRGKQCPFKRVVKKCCDGFKGPRCNQPEKVENVKPNNETMIPHHKLPFATCVLWGKDHFRTFSG
TFFQFNGNCEYKLSGSSSWQVNVVNDACHDWDTCTKTLKMTFGSIELTAVGKRVTIDNVKLQDHENLHKDGVIIE
RKGDYTYLKFSDGVRLKWSSDSFNIFVTVDEQYMNKVSGLCGDYNNNLNNDLLLPDDSYTNEPSIFGNSWRLDSSC
NEAAPSNNPCNTPALEEEARDACFLIDNPSDSFRLCRKVVNASRYLEQCKKDHCSASKSIKDPERSKRLALCNSFAA
MAAECSENFVNTEWRKANRCPKTCPGEKVYTECATSCPKTCQNKHQNFAGTECKQECSPGCVCRDGFYLDAGHN
NTCVKAEDCTCSFRGNFYSTGQKVAVECNECVCNGGKWTCTDRKCPRTCSVIGMGHYRTFDGKHYDFKGNCEYTL
VEQIDESINPKLYISHRINHALLKDGPTELIIKSMKTIVSLKQNIIYINSQLVSQLPYGNSDVLVRKASDFFFTVEGKGFKV
LFDGVRIYITLDPFFVDNVRGLCGTYNFKSADDFFPPSGFIESDVVSFVDSYKVDGYCLTNNQNQPCENYIAAEAEAS
RLCNTLRDHNVFKNCESVVDVASFIEACKYDLCSDGNQLHRDLFRCRAIAAYAHECAAKGIVIDWLDHDDLREIKSA
CYHSNYGKCYGGSTYSECSRSFNTTCRDLSSKNREKFSNQNINYCVAGCTCPENQYFENINGNLQCVAKESCSCYD
MSSSKYYAAGEKIKKACSTCTCFGGEWRCDNIECKDIIKCPNNLVFSKNASTCPKTCGNKDHYRDCDMEAEGCKCP
NGTILDYNMRCVPEKECPCRHGGKVHNHLEKIKVRCNLCECNGGHWSCSSQKCDGVCISAGDPHYTTFDGLRFSY
QGNCKYVLSQTENQKFRVVAENVPCGTSGVTCTKNIFIQYQDLTINLMRGRNIEVNGVELANLEQGARVFGDVHI
MVAGLYHIVNSTDFLIKWDGATRLYIVVHSQWKGKLQGICGNFDM**DSSNDLI**TVSGILGSYQELVESWKVEESCTV
DSNPFIDESDPCHGHLHRKEWATKECSMINTPSADNPFTPCLAKLDSTDLIKSHIECLYDACSCDKGGDCECLCSSLA
AFSELCIKAGVPIKWRSLHKCPIQCEYGKEYLPCGPICQQTCMDLSTGNNPQCNDAGCVEGCFCPAGTVADYNGRC
IEPANCDCYLDNNRYPVGSQITKDCLLCECRNGSFDCSQNIADCKPKCDLKTEFTCPSDKTCIPKEWLCDKVNDCGD
NSDELNCKCDVTNKTFVCNNGQCIDKKYLCDGM**PNCRDGSDE**DNCKPTCSEFQCENNKCIPFSWTCDGYPDCGF
LDESDEMNCNKTSNCTDDVREFKCNKDSECLPITEKCDGHDDCGDGSDERHCRCICEKGFACKSSCECIDPKRVCDS
VRDCSDGSDEKGCSCAHDEYRCNGGKCINATLLCDGKKHCPNGDDESHPKCTTTTTTSTSTTTTTTTTGQPAVVTT
VTVISNQTSTSTETPAISSTTTTSQCSPELYQCKENPTVCINKTQLCDQTCDCGFNCLDESNCYSTTSGPCTTFLCDINS
GTTRKCLDPRRLCDGHQDCFDNTDEDPSRCFETTTTSTTVPPPNYYCDHGIEISNITTLNHTLMNGHELDGVTDEID
NFFSENKKLVVPSESFNGVRFDFFTQVHITRVLFNMVNKAFITVKIDNDVDLAIRKVNIDDTNSIIIAEMNPELVNEIS
TRSLTITIQAFDEDVKVSKLTIEGCYDYDYNGGAGTTTVTGYTRATRPTRQTTKITT
KTGSTTTPGECEETEIIGNPKYVSDIETTPFVFPADLIPNSGGIELKKLPAKIKVIVTTGIEIPIISIEFSNKKTNIKEYTIELID
RNGKTIYEDKENKLITKINSENPATEIIITILSTKDNQYPKFVELSIKACKHYDIHQTTTNVFTSFTTKEQYCEKELGLRNN
IISSDDIVLSSKKGNVNEIRLGYKKLWKAAKDDLLPSVKVFFNEPVELSGLKIRGTVDRIKIMYRTEDLENFEYIQSEDFK
RLPAELNGESTIKFPRVTDVKEVIVDFFVSFIGEPVTAQIELLGCGEIVDHTRITTKRPVCENRDILLVDEAVDLINNQA
DEEIDIHEIADNNQTGWKPTILSDYIDIKLMNAIDIVKIVVLPGSNVKKYKLVYKDINEVEKDTQKNEKSEPIVYYFPRD
VDLIKTIRFKPIKKFNPNQQYNIKLKIFGCIKSRNYTTKTKPTTKLSTISTTTETQECESQDILINPASIEEINNQKDEDIHF
EVISSHYPKHKGWKQQIKSDFVDLKLANALKIVKFEVVNGSNVKKYYLRMQDVNEKSHEYLKVEDSDFIVKKNIDLV
RVIRFKPLKKINKKQDYNIKFKIYACTTFKTHPEQSKTSTSTISTTTTTTTTTVSSATNKETSVYHTDVTSHGTTKTSHGT
TIASHGTTKTTHKTLYTTQKYCENNDVLLYPENVEEINNQEDEEVPLGQISDNNEIPGWVPHRRSDFVDIRLAHALRII
KLVVVNTSNVKNFIVKLQDVELKIHTLKGNGESELLIPTDLDYIKVIRFHPKIKPEQDLKSFHIKLKLIACTKPRTVPTKKT
TKKITTFTTKTKESCELYDVLSQLDNKNYEILSVNTIKPVKNIKKLEINSGEELRIKFEGIEKIVKIKVDKNSNIKSFFIKYVN
SKNDLINLEKYEDEIVYLPYIEMREIIITPLTKWKNNEKFKFDIDISGCKKGKQQRRKM

Uncharacterized *Euchlanis dilatata*-specific protein

rf 1 ctg.003923F.g152291.t1

MW: 284572

(Datki et al., 2023)

MDKEQPIFTITSSVPRDRRIEDIYPDSARPYDAPRGTNTVTFTFNLPELKTAEVGLVEILPGSNVDTVKVTTLRDTEPV
DEKETNDNQPIIGFIFTSINEIRITLTTDKPTLKDVRIRILVCLHIYTTTTSTTGEPASTTTVIVSSTTTPTTIITPVIITTRKTD
KPETTTTRPTGTTTKTSTTKPTGTTTKTSTTRPTGTTTTTTRPTGTTTSTTRPTGTTTTTTTTKPTGTTTTTTTTKPTGTT
TTTSTTKPTGTTTTTTRTEPITTTRISTKHTHQTDGITTTTTNDEDTTTTLTEPTTTSTNEEETTTTTTTESTTTSTNEEDT
TTTTTEPTTTSTVTVPTTTTTITPICTKTDGMDKEQPIFTITSSVPRDRRIEDIYPDSARPYDAPRGTNTVTFTFNLPELK
TAEVGLVEILPGSNVDTVKVTTLRDTEPVDEKETNDNQPIIGFTFTSINEIRITLTTDKPTLKDVRIRILVCLHIYTTTTSTT
GEPASTTTVIVSSTTTPTTTITPVIITTRKTDKPETTTTRTTGKTTSTTRPTGTTTKTSTTKPTGTTTKTSTTKPTGTTTKTS
TTRPTGTTTTTTRPTGTTTSTTRPTGTTTTTTTTKPTGTTTTTTTTKPTGTTTTTSTTKPTGTTTTTTRTEPITTTRISTKH
THQTDGITTTTTNDEDTTTTLTEPTTTSTNEEETTTTTTTESTTTSTNEEDTTTTTTEPTTTSTVTVPTTTTTITPICTKTD
GMDKEQPIFTITSSVPRDRRIEDIYPDSARPYDAPRGTNTVTFTFNLPELKTAEVGLVEILPGSNVDTVKVTTLRDTEP
VDEKETNDNQPIIGFIFTSINEIRITLTTDKPTLKDVRIRILVCLHIYTTTTSTTGEPASTTTVIVSSTTTPTTIITPVIITTRKT
DKPETTTTRTTGKTTSTTRPTGTTTKTSTTKPTGTTTKTSTTKPTGTTTKTSTTRPTGTTTTTTRPTGTTTSTTRPTGTTT
TTTTTKPTGTTTTTTTTKPTGTTTTTSTTKPTGTTTTTTRTEPITTTRISTKHTHQTDGITTTTTNDEDTTTTLTEPTTTST
NEEETTTTTTTESTTTSTNEEDTTTTTTEPTTTSTVTVPTTTTTITPICTKTDGMDKEQPIFTITSSVPRDRRIEDIYPDSA
RPYDAPRGTNTVTFTFNLPELKTAEVGLVEILPGSNVDTVKVTTLRDTEPVDEKETNDNQPIIGFTFTSINEIRITLTTD
KPTLKDVRIRILVCLHIYTTTTSTTGEPASTTTVIVSSTTTPTTTITPVIITTRKTDKPETTTTRTTGKTTSTTRPTGTTTKTS
TTKPTGTTTKTSTTKPTGTTTKTSTTRPTGTTTSTTRPTGTTTTTTTTKPTGTTTTTTTTKPTGTTTTTSTTKPTGTTTTT
TRTEPITTTRISTKHTHQTDGITTTTTNDEDTTTTTTEPTTTSTVTVPTTTTTITPICTKTDGMDKEQPIFTITSSVPRDR
RIEDIYPDSARPYDAPRGTNTVTFTFNLPELKTAEVGLVEILPGSNVDTVKVTTLRDTEPVDEKETNDNQPIIGFTFTSI
NEIRITLTTDKPTLKDVRIRILVCLHIYTTTTSTTGEPASTTTVIVSSTTTPTTTITPVIITTRKTDKPETTTTRTTGTTTSTTR
PTGTTTKTSTTRPTGTTTKTSTTRPTGTTTTTTRPTGTTTSTTRPTGTTTTTTTTKPTGTTTTTTTTKPTGTTTTTSTTKP
TGTTTTTTRTEPITTTRISTKHTHQTDGITTTTTNDEDTTTTLTEPTTTSTNEEETTTTTTTESTTTSTNEEDTTTTTTEPT
TTSTVTVPTTTTTITPICTKTDGMDKEQPIFTITSSVPRDRRIEDIYPDSARPYDAPRGTNTVTFTFNLPELKTAEVGLVE
ILPGSNVDTVKVTTLRDTEPVDEKETNDNQPIIGFTFTSINEIRITLTTDKPTLKDVRIRILVCLHIYTTTTSTTGEPASTTT
VIVSSTTTPTTTITPVIITTRKTDKPETTTTRTTGKTTSTTRPTGTTTKTSTTKPTGTTTKTSTTKPTGTTTKTSTTRPTGTT
TTTTRPTGTTTSTTRPTGTTTTTTTTKPTGTTTTTTTTKPTGTTTTTSTTKPTGTTTTTTRTEPITTTRISTISTTSHFCETF
EIMGNPIYITDIQTVPFIFPELLSPLSGGANFTKPSAEIVVLFTSGNEIPINSIQFSTFSNIKKFTVQIVSTDNSIIEFVTKQT
NEPVYVQSNKKISKIVINILSTTDSRNPSNVELSIKSCTHITLATKLTSAHPASTTTVIVSSTTKPTSTISPVITSSTSTLSQF
TTTHSCEMFEIMGNPMYISDIQTEPFIFPDLFVPSSGGANFSTPTSDLIVSFTLGNEVPLEIIEFSPLSNIKQFIIKLISND
NELIETVSKQKNEPVYVKTNKKISKIVINILSTTDNKYPQNVELSIKSCTHIELTTKPVSTTTFTYTTTTFENCIYSSWSQW
TSCSSECGHSTKSRNRYVISGICHGELHQSKDCDNVNCSCVFTAEVFVTLTRRQVPIDLVVGYIKKSPNSPVSSTDPAV
SIFDVVPFGTHIHVDNCSEIICDKNGLQIIHHNCSSIFFFRILFLYFILMLLIILKRIVPGLNGLHGQFVVQIVMHLVYNTE
LENQVRILLMYAMNLH

Suppl. Figure S1. Peptides derived from rotifer-specific proteins. The R-SSPO/1 (derived peptides: DSSNDL and PNCRDGSDE) and uncharacterized protein (derived peptide: STTRPTGTT) types of synthesized peptides are highlighted in color, while the theoretical but untested versions (TEDLENFEYIQSEDFK, CTKTLKMTF, RNIEVNGVE, and IPTDLDYIKV) are underlined.
