# Supplementary material for: Protective Effect of a Hexapeptide Derived from Rotifer-Specific SCO-Spondin Against Beta-Amyloid Toxicity
Source: Int J Mol Sci. 2025 May 26;26(11):5109. doi: 10.3390/ijms26115109 (PMC12154537; doi:10.3390/ijms26115109)
Supplement: Supplementary file 1 [file ijms-26-05109-s001.zip › Suppl Figure S2.pdf]

Suppl. Figure S2

The HPLC Chromatogram and ESI-MS Spectrum of Purified Peptides Derived from Rotifer-Specific Proteins

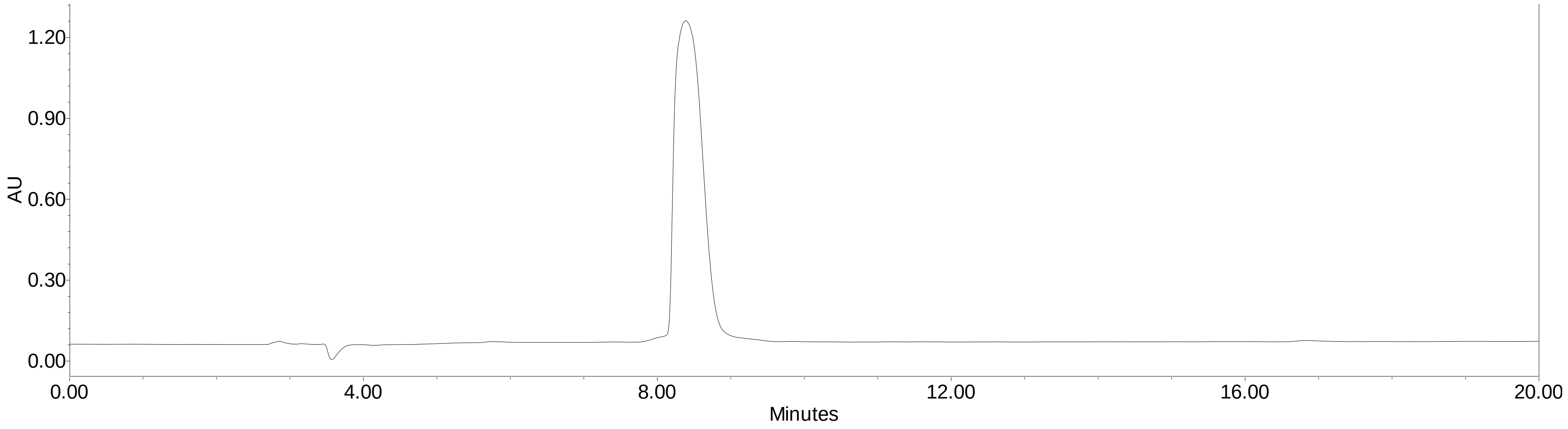

Figure A1: HPLC chromatogram of purified PNCRDGSDE. Column: MZ-Analytical PerfectSil C18, 250x4.6 mm, Gradient: 5-25% solvent B in solvent A over 20 min, Flow rate: 1 mL/min, wavelength: 220 nm, solvent A: 0.1% TFA, solvent B: 0.1% TFA/80% acetonitrile

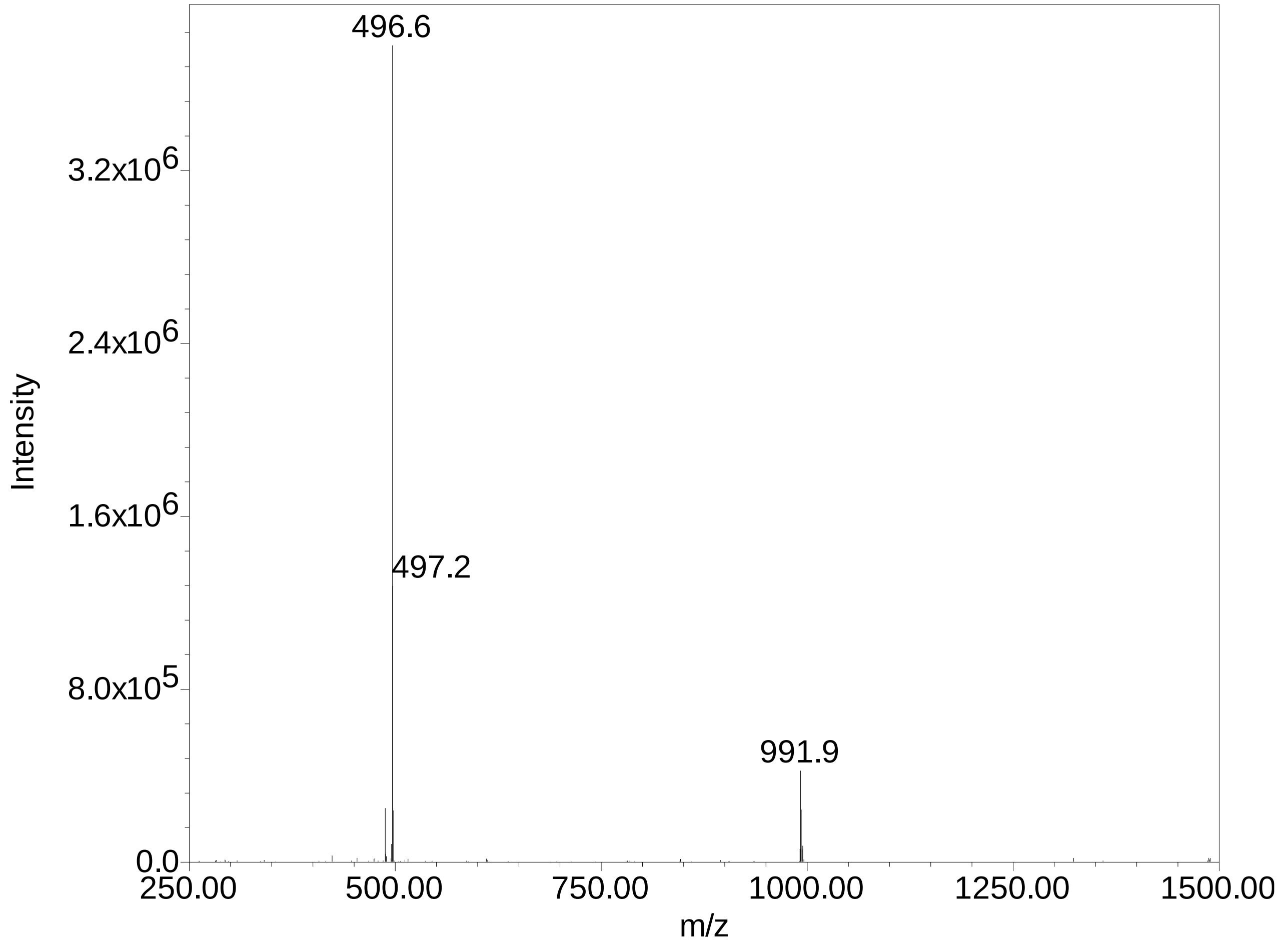

Figure A2: ESI-MS spectrum of purified PNCRDGSDE.

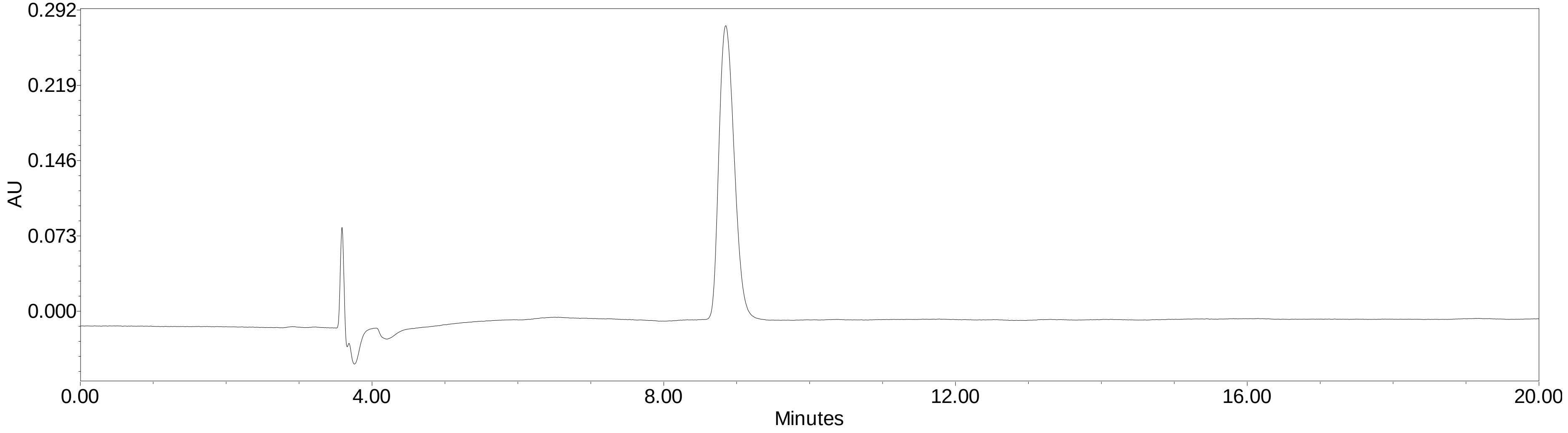

Figure B1: HPLC chromatogram of purified STTRPTGTT. Column: MZ-Analytical PerfectSil C18, 250x4.6 mm, Gradient: 5-25% solvent B in solvent A over 20 min, Flow rate: 1 mL/min, wavelength: 220 nm, solvent A: 0.1% TFA, solvent B: 0.1% TFA/80% acetonitrile

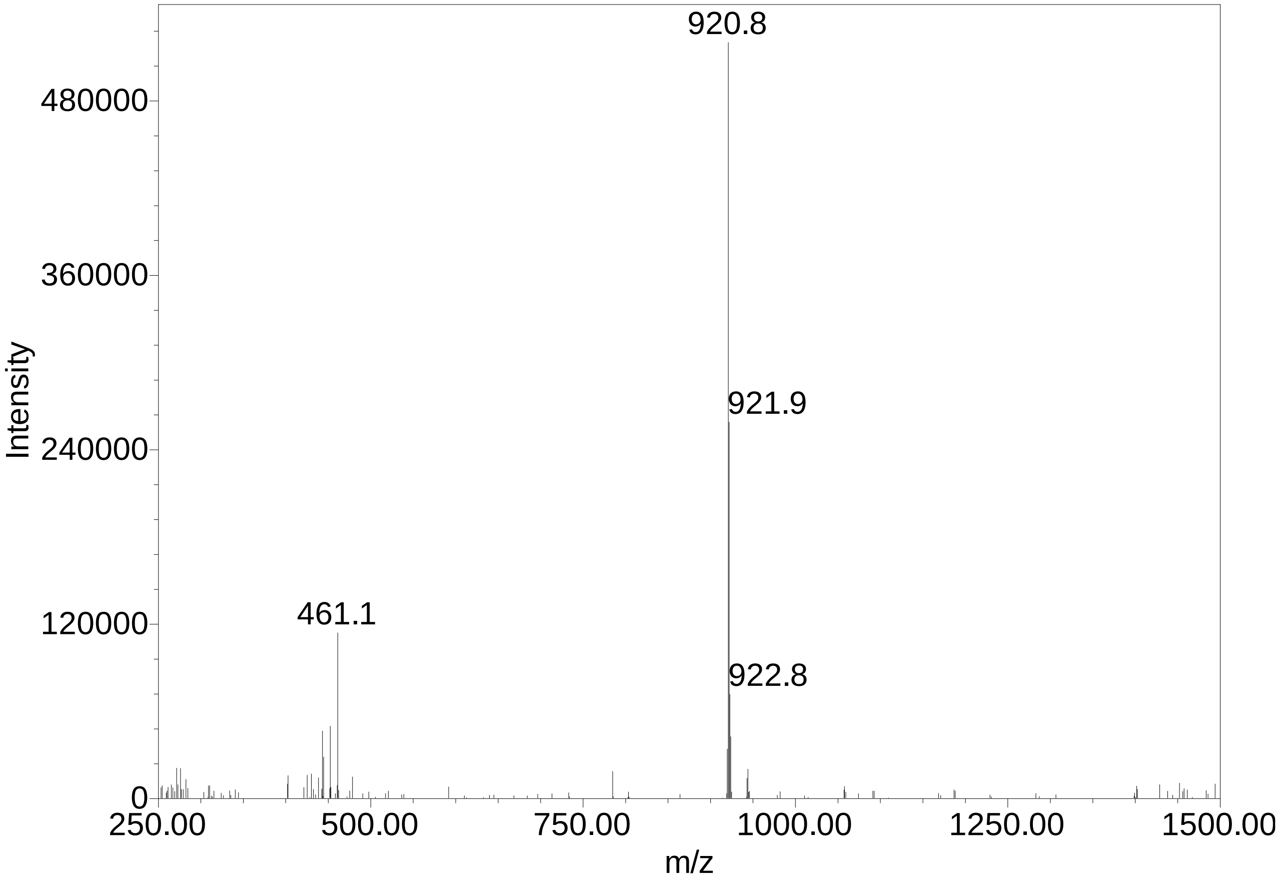

Figure B2: ESI-MS spectrum of purified STTRPTGTT.

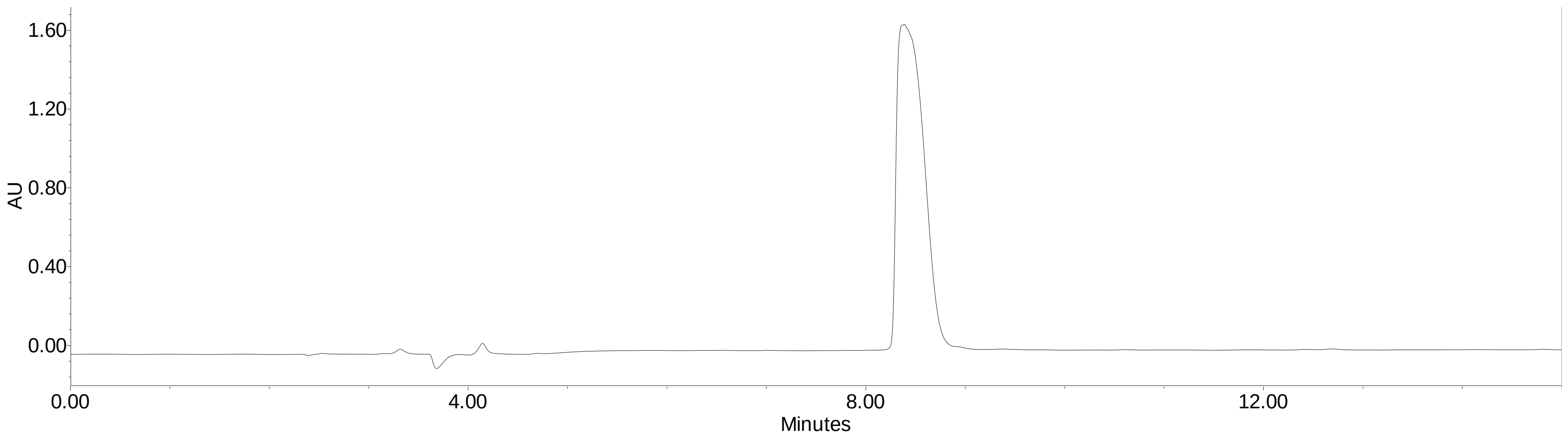

Figure C1: HPLC chromatogram of purified DSSNDL. Column: MZ-Analytical PerfectSil C18, 250x4.6 mm, Gradient: 10-25% solvent B in solvent A over 15 min, Flow rate: 1 mL/min, wavelength: 220 nm, solvent A: 0.1% TFA, solvent B: 0.1% TFA/80% acetonitrile

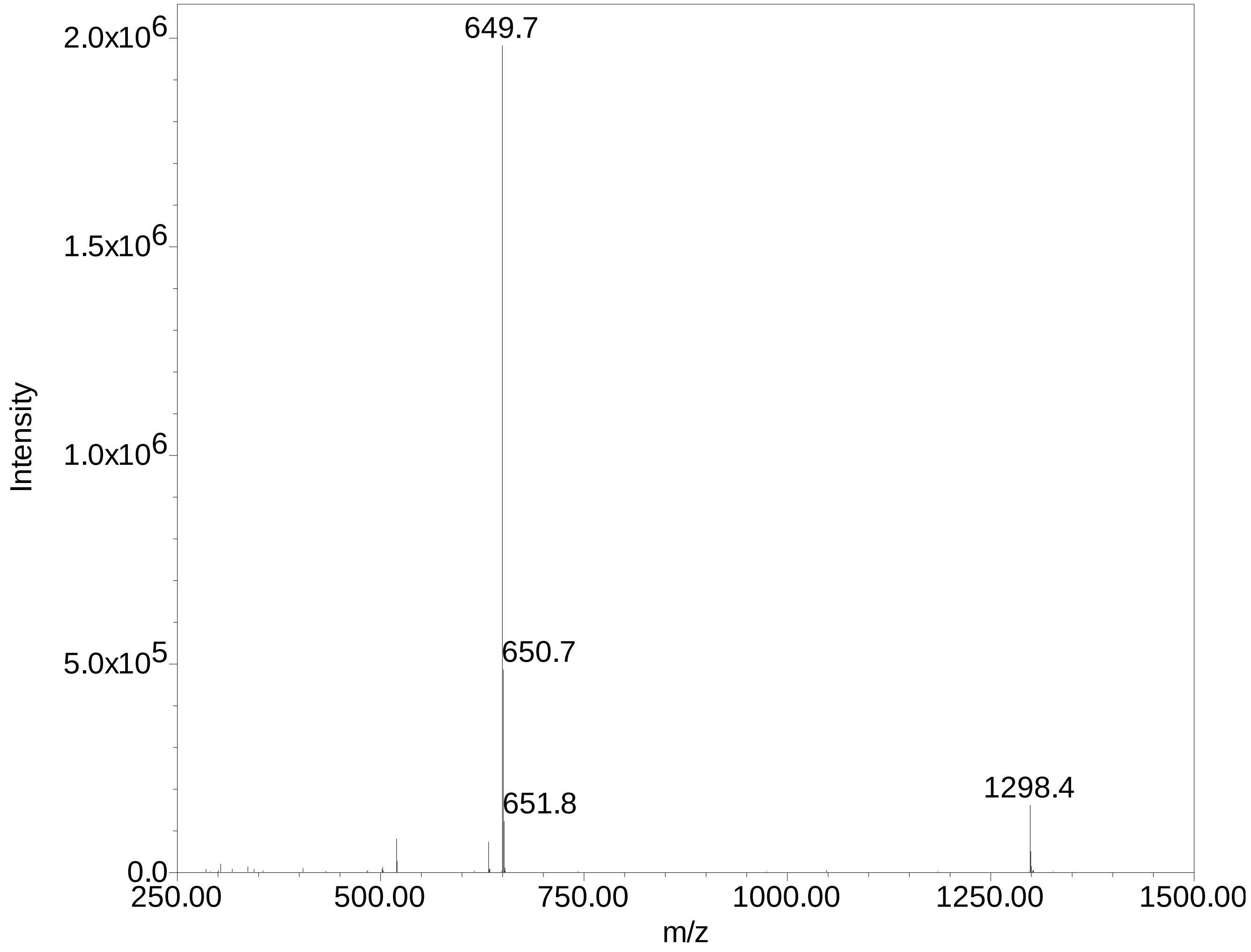

Figure C2: ESI-MS spectrum of purified DSSNDL.
