# Supplementary material for: Protective Effect of a Hexapeptide Derived from Rotifer-Specific SCO-Spondin Against Beta-Amyloid Toxicity
Source: Int J Mol Sci. 2025 May 26;26(11):5109. doi: 10.3390/ijms26115109 (PMC12154537; doi:10.3390/ijms26115109)
Supplement: Supplementary file 1 [file ijms-26-05109-s001.zip › Suppl Table S1.pdf]

Suppl. Table S1. The presence of R-SSPO/1-derived peptides in rotifer and human proteome database

DSSNDL

Scientific Name	No. Hits	Avg. Total Score	Avg. Query Cover (%)	Avg. Per. Identity	Avg. E value
Adineta ricciae	6	27.73	73.33	100.00	94.00
Adineta vaga	30	22.77	75.43	90.64	80.77
Brachionus calyciflorus	1	22.70	66.00	100.00	84.00
Brachionus koreanus	4	31.50	77.00	85.71	77.00
Brachionus plicatilis	13	24.07	74.46	91.42	74.38
Rotaria magnacalcarata	19	22.70	66.00	100.00	84.79
Rotaria socialis	2	44.85	77.00	77.78	120.00
Rotaria sordida	2	43.30	100.00	90.00	0.31
Rotaria sp. Silwood1	14	22.56	69.93	94.90	97.21
Rotaria sp. Silwood2	6	22.50	77.00	87.50	102.00
Seison nebaliae	3	22.70	77.00	86.90	84.67

PNCRDGSDE

Scientific Name	No. Hits	Avg. Total Score	Avg. Query Cover (%)	Avg. Per. Identity	Avg. E value
Adineta ricciae	8	89.00	95.50	93.06	0.27
Adineta vaga	3	101.80	94.00	100.00	0.16
Brachionus calyciflorus	1	89.50	100.00	88.89	0.31
Didymodactylos carnosus	3	123.00	100.00	88.89	0.31
Rotaria magnacalcarata	29	75.03	100.00	95.24	0.10
Rotaria socialis	24	215.22	100.00	92.13	0.20
Rotaria sordida	16	101.00	100.00	94.82	0.08
Rotaria sp. Silwood1	17	85.76	100.00	95.43	0.07
Rotaria sp. Silwood2	13	95.61	100.00	100.00	0.02
Seison nebaliae	2	93.40	94.00	94.44	0.23

STTRPTGTT

Scientific Name	No. Hits	Avg. Total Score	Avg. Query Cover (%)	Avg. Per. Identity	Avg. E value

Adineta ricciae	20	53.00	92.20	85.83	30.13
Adineta steineri	17	137.03	95.06	93.63	24.99
Adineta vaga	2	45.30	100.00	87.50	29.00
Brachionus calyciflorus	3	175.87	100.00	88.43	19.67
Brachionus plicatilis	1	190.00	100.00	87.50	29.00
Didymodactylos carnosus	2	24.00	77.00	100.00	29.50
Rotaria magnacalcarata	9	101.13	96.00	100.00	1.80
Rotaria socialis	10	146.42	100.00	100.00	1.80
Rotaria sordida	13	27.87	93.54	77.99	43.54
Rotaria sp. Silwood1	18	368.11	98.72	79.11	51.22
Rotaria sp. Silwood2	5	41.60	88.20	90.00	41.20

TEDLENFEYIQSEDFK

**Scientific Name**	**No. Hits**	**Avg. Total Score**	**Avg. Query Cover (%)**	**Avg. Per. Identity**	**Avg. E value**
Adineta ricciae	2	28.20	50.00	87.50	3.40
Adineta steineri	10	62.65	70.40	70.13	6.73
Adineta vaga	3	37.87	58.33	80.56	7.67
Brachionus calyciflorus	15	38.77	72.53	68.88	6.61
Brachionus plicatilis	6	29.06	73.40	67.53	3.87
Brachionus rubens	1	44.10	93.00	58.82	6.70
Didymodactylos carnosus	1	26.50	93.00	45.83	13.00
Rotaria magnacalcarata	7	27.01	62.00	71.43	9.54
Rotaria socialis	15	26.93	62.00	72.78	9.95
Rotaria sordida	9	27.26	83.00	61.61	7.80
Rotaria sp. Silwood1	18	28.15	68.50	69.43	5.28
Rotaria sp. Silwood2	11	27.91	71.91	70.94	5.70
Seison nebaliae	3	27.07	72.67	72.08	10.13

CTKTLKMTF

Scientific Name	No. Hits	Avg. Total Score	Avg. Query Cover (%)	Avg. Per. Identity	Avg. E value
Adineta ricciae	6	27.73	73.33	100.00	94.00
Adineta steineri	30	22.77	75.43	90.64	80.77
Adineta vaga	1	22.70	66.00	100.00	84.00
Brachionus plicatilis	4	31.50	77.00	85.71	77.00
Didymodactylos carnosus	13	24.07	74.46	91.42	74.38
Rotaria magnacalcarata	19	22.70	66.00	100.00	84.79
Rotaria socialis	2	44.85	77.00	77.78	120.00
Rotaria sordida	2	43.30	100.00	90.00	0.31
Rotaria sp. Silwood1	14	22.56	69.93	94.90	97.21
Rotaria sp. Silwood2	6	22.50	77.00	87.50	102.00
Seison nebaliae	3	22.70	77.00	86.90	84.67

RNIEVNGVE

Scientific Name	No. Hits	Avg. Total Score	Avg. Query Cover (%)	Avg. Per. Identity	Avg. E value
Adineta ricciae	12	41.27	87.67	89.73	59.50
Adineta steineri	13	38.13	82.54	92.31	59.69
Adineta vaga	3	44.90	88.67	90.47	59.67
Brachionus calyciflorus	2	26.50	88.50	87.30	29.61
Brachionus plicatilis	3	31.93	88.33	83.20	34.41
Didymodactylos carnosus	5	27.24	90.80	80.95	41.40
Rotaria magnacalcarata	6	49.45	88.50	85.71	60.00
Rotaria socialis	14	45.01	100.00	83.10	55.29
Rotaria sordida	7	56.20	100.00	85.71	60.00
Rotaria sp. Silwood1	26	39.39	93.54	79.94	42.46
Rotaria sp. Silwood2	6	57.95	100.00	85.71	59.83
Seison nebaliae	3	23.23	77.00	85.71	54.00

IPTDLDYIKV

Scientific Name	No. Hits	Avg. Total Score	Avg. Query Cover (%)	Avg. Per. Identity	Avg. E value
Adineta ricciae	5	26.16	72.00	97.50	7.12
Adineta steineri	12	25.98	75.00	92.06	10.16
Adineta vaga	1	24.00	70.00	85.71	36.00
Brachionus calyciflorus	1	71.30	100.00	61.54	13.00
Brachionus manjavacas	1	25.20	80.00	87.50	13.00
Brachionus plicatilis	2	26.05	85.00	82.64	8.05
Rotaria magnacalcarata	23	27.73	76.96	88.92	14.26
Rotaria socialis	19	28.37	70.00	89.47	24.00
Rotaria sordida	16	25.59	70.62	93.97	15.64
Rotaria sp. Silwood1	10	27.50	75.00	94.03	13.75
Rotaria sp. Silwood2	9	26.80	72.50	97.22	3.48
Seison nebaliae	2	24.40	80.00	82.85	26.00

Suppl. Table. Summary of the first 100 protein BLAST hit of the peptide searches in *Rotifera* (taxid:10190, date: 08.03.2024).

DSSNDL

Description	Total Score	Query Cover (%)	Per. Identity	E value
immunoglobulin light chain junction region	21.40	100.00	100.00	28.00
immunoglobulin heavy chain junction region	18.90	100.00	83.33	297.00
SCO-spondin (precursor)	18.90	100.00	83.33	298.00
TPA: SCO-spondin precursor	18.90	100.00	83.33	298.00
hCG16178	18.90	100.00	83.33	298.00
1-phosphatidylinositol 3-phosphate 5-kinase	33.90	100.00	83.33	299.00
signal-induced proliferation-associated 1-like protein 3	18.90	100.00	83.33	299.00
phosphoinositide kinase, FYVE-type zinc finger containing	33.90	100.00	83.33	299.00
KIAA0545 protein	18.90	100.00	83.33	299.00
KIAA2036 protein	18.90	100.00	83.33	299.00

PNCRDGSDE

Description	Total Score	Query Cover (%)	Per. Identity	E value
transmembrane protease serine 6	64.70	100.00	88.89	0.05
PVAE354	64.70	100.00	88.89	0.05
matriptase-2	64.70	100.00	88.89	0.05
very low density lipoprotein receptor	148.00	100.00	77.78	0.78
SCO-spondin	198.00	100.00	77.78	1.60
TPA: SCO-spondin precursor	198.00	100.00	77.78	1.60
hCG16178	214.00	100.00	77.78	1.60
sortilin-related receptor preproprotein	176.00	88.00	100.00	1.60
gp250 precursor	176.00	88.00	100.00	1.60
sortilin related receptor 1	176.00	88.00	100.00	1.60

STTRPTGTT

Description	Total Score	Query Cover (%)	Per. Identity	E value
immunoglobulin heavy chain junction region	22.30	88.00	87.50	29.00
target of Nesh-SH3	54.10	100.00	87.50	37.00
ABI family member 3 binding protein	54.10	100.00	87.50	37.00
ABI family, member 3 (NESH) binding protein	22.30	88.00	87.50	37.00
ABI gene family, member 3 (NESH) binding protein	22.30	88.00	87.50	37.00
immunoglobulin light chain junction region	21.40	66.00	100.00	46.00
mucin-19 precursor	22.05	100.00	66.67	53.00
E3 ubiquitin-protein ligase NEDD4	21.8	100.00	77.78	53.00
NEDD4 protein	21.8	100.00	77.78	53.00
NEDD4 E3 ubiquitin protein ligase	21.8	100.00	77.78	53.00

TEDLENFEYIQSEDFK

Description	Total Score	Query Cover (%)	Per. Identity	E value
zinc finger protein	27.80	81.00	55.56	1.50
Chain A, Tyrosine-protein kinase HCK	27.40	56.00	72.73	2.10
Chain A, HAEMATOPOETIC CELL KINASE HCK	27.40	56.00	72.73	2.10
HCK protein	27.40	56.00	72.73	2.10
Peptidase M20 domain containing 1	43.70	62.00	87.5	2.90
N-fatty-acyl-amino acid synthase/hydrolase PM20D1 precursor	43.70	62.00	87.5	2.90
KIF14 protein	43.30	81.00	66.67	3.00
PXDN protein	26.50	93.00	48.00	4.20
peroxidasin homolog precursor	26.50	93.00	48.00	4.20
melanoma-associated antigen MG50	26.50	93.00	48.00	4.20

CTKTLKMTF

Description	Total Score	Query Cover (%)	Per. Identity	E value
catsper channel auxiliary subunit epsilon	25.20	100.00	77.78	3.20
hCG2036691	25.20	100.00	77.78	3.20
hCG2042312	25.20	100.00	77.78	3.20
immunoglobulin heavy chain junction region	23.50	88.00	75.00	8.40
intermembrane lipid transfer protein VPS13A	40.30	88.00	100.00	26.00
vacuolar protein sorting 13 homolog A	40.30	88.00	100.00	26.00
chorea-acanthocytosis	40.30	88.00	100.00	26.00
vacuolar protein sorting 13A (yeast),	40.30	88.00	100.00	26.00
KIAA0986 protein	22.70	66.00	100.00	26.00
ALS2CR11	21.40	77.00	85.71	75.00

RNIEVNGVE

Description	Total Score	Query Cover (%)	Per. Identity	E value
PARD3 protein	23.10	100.00	77.78	18.00
par-3 family cell polarity regulator	23.10	100.00	77.78	18.00
SE2-5L16 protein	23.10	100.00	77.78	18.00
SE2-5LT1 protein	23.10	100.00	77.78	18.00
SE2-5T2 protein	23.10	100.00	77.78	18.00
CTCL tumor antigen se2-5	23.10	100.00	77.78	18.00
SH3 and multiple ankyrin repeat domains protein 3	21.80	66.00	100.00	53.00
Na(+)/H(+) exchange regulatory cofactor NHE-RF3	42.40	77.00	100.00	53.00
PDZ domain containing-protein	42.40	77.00	100	53.00
kinase D-interacting substrate	36.90	100.00	77.78	75.00

IPTDLDYIKV

**Description**	**Total Score**	**Query Cover (%)**	**Per. Identity**	**E value**
immunoglobulin heavy chain junction region	26.90	70.00	100.00	0.55
hypothetical protein G5576_006393	41.60	100.00	61.54	2.80
hypothetical protein KI723_111566	41.60	100.00	61.54	2.80
mediator complex subunit 17	41.60	100.00	61.54	2.80
vitamin D3 receptor interacting protein	41.60	100.00	61.54	2.80
mediator of RNA polymerase II transcription subunit 17	41.60	100.00	61.54	2.80
thyroid hormone receptor-associated protein complex component TRAP80	41.60	100.00	61.54	2.80
cyclic nucleotide-gated cation channel alpha-3	25.20	100.00	70.00	3.90
Chain A, Cyclic nucleotide-gated cation channel alpha-3	25.20	100.00	70.00	3.90
dystroglycan 1	23.50	100.00	60.00	16.00

Suppl. Table. Summary of 10 representative protein BLAST hit of the peptide searches in *Homo sapiens* (taxid:9606, date: 08.03.2024).
